# Novel perspective on contractile properties and intensity‐dependent verification of force–frequency relationship during neuromuscular electrical stimulation

**DOI:** 10.14814/phy2.14598

**Published:** 2020-11-23

**Authors:** Aya Tomita, Shuhei Kawade, Toshio Moritani, Kohei Watanabe

**Affiliations:** ^1^ Laboratory of Neuromuscular Biomechanics Faculty of Liberal Arts and Sciences and School of International Liberal Studies Chukyo University Nagoya Japan; ^2^ MTG Co., Ltd. Nagoya Japan; ^3^ Faculty of Sociology Kyoto Sangyo University Kyoto Japan

**Keywords:** contractile properties, neuromuscular electrical stimulation, stimulus frequency, stimulus intensity

## Abstract

**Purpose**

The aims of the present study were: (a) to examine the effect of the stimulus intensity on force‐frequency and torque fluctuation–frequency relationships during Neuromuscular electrical stimulation; and (b) to identify a novel parameter that can be used to evaluate muscle contractile properties. Methods: Electrically elicited joint torque involving the quadriceps femoris muscle was recorded during neuromuscular electrical stimulation at two different stimulus intensities in 19 healthy men. Stimulation frequencies were set at 5–40 Hz with a duration of 10 s. Evoked joint torque was compared among all stimulation frequencies between the two stimulus intensities (68 and 113 V). The torque fluctuation at each stimulation frequency as the change in the contraction pattern was also compared between the intensities. Torque and torque fluctuation were normalized at each frequency by the largest torque or torque fluctuation, respectively. We extracted a novel parameter: the arrival point of tetanic contraction based on force‐frequency and torque fluctuation‐frequency curves. Results: There were significant differences in normalized torque at 5–25 and 40 Hz and in normalized torque fluctuation at 15–30 and 40 Hz between the two stimulus intensities. Extracted parameters showed no significant difference between the intensities. Conclusion: The results suggest that force–frequency relationships during neuromuscular electrical stimulation are influenced by the intensity of stimulation applied to the quadriceps femoris muscle. However, we consider that it is possible to simultaneously evaluate contractile properties using the novel parameter.

## Graphical abstract

We tested the effect of stimulus intensity on force‐frequency relationship and torque fluctuation‐frequency relationship during neuromuscular electrical stimulation (NMES), and proposed new parameter to evaluate muscle contractile properties. We suggest that the stimulus intensity should be considered on investigating the force‐frequency relationship during NMES in the quadriceps femoris muscle. However, we suggest that simultaneously we can evaluate contractile properties with using new parameters.

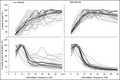

## 1 INTRODUCTION

Neuromuscular electrical stimulation (NMES) has been used as an effective training or rehabilitation tool (Bochkezanian et al., 2018; Bremner et al., 2017; Maffiuletti, 2010; Nussbaum et al., 2017). Various parameters are related to NMES, such as the stimulus intensity, stimulus frequency, pulse width, duty cycle, duration, electrode location, and electrode size (Glaviano & Saliba, 2016). Among them, the stimulus intensity and stimulus frequency are directly related to the development of evoked torque. It is well‐known that physiological responses during NMES depend on the stimulus intensity. With a higher stimulus intensity, discomfort increases and fatigue is induced faster (Binder‐Macleod et al., 1995; Delitto et al., 1992).

The muscle contraction pattern changes from twitch contraction, to incomplete tetanus, and then complete tetanus with an increase in the stimulation frequency during NMES. The force–frequency curve shows this transition of the contraction pattern during NMES. Also, the torque fluctuation of the developed torque may slow down when the contraction pattern reaches the tetanic phase. The contractile properties of skeletal muscle differ according to aging and fatigue (Allman & Rice, 2004; Roos et al., 1999). The force–frequency relationship, showing one aspect of the contractile properties, is a changeable characteristic, and there are individual differences in the stimulation frequency causing tetanic contraction (Binder‐Macleod et al., 1998). To clarify individual differences and associated factors, examination of the force–frequency relationship using NMES should be adopted in a wide range of subjects (e.g., differences in age, sex, and level of disability). The relationship between the evoked torque on NMES and stimulation frequency (force–frequency relationship) has been applied to evaluate muscle contractile properties. Fragala et al. (2015) described this basic contractile element (as shown in the force–frequency relationship) as an indicator of muscle quality.

Muscle contractile properties can be evaluated based on the force–frequency relationship using NMES (Binder‐Macleod & McDermond, 1992; Kirk et al., 2018; Kirk & Rice, 2017). Some previous studies employed high stimulus intensities during NMES to evaluate the contractile properties of human skeletal muscles (Allman & Rice, 2004; Doucet et al., 2012; Soo et al., 1988). Meanwhile, Binder‐Macleod et al. (1995) reported that twitch contractile speeds did not differ among NMES intensities of 20% of maximal voluntary isometric contraction (MVC), 50% MVC, and 80% MVC. However, they also reported that the force–frequency relationship did not differ when torque was evoked at 20% MVC and 50% MVC at 20–70‐Hz stimulation frequencies, while 80% MVC elicited a slight leftward shift in response to both evoked contraction levels. In some previous studies, it was suggested that the motor unit recruitment order during NMES is nonselective, and recruited motor units are not related to the “size principle” (Binder‐Macleod et al., 1995; Gregory & Bickel, 2005; Jubeau et al., 2007). In another study, NMES could recruit both slow and fast muscle fibers regardless of the contraction level, resulting in increased muscle strength and muscle hypertrophy of both muscle fiber types at high and low intensities after 8‐week NMES training (Natsume et al., 2018). If the motor unit recruitment order on NMES is completely randomized, the force‐frequency curve should not vary depending on the stimulus intensity. As mentioned above, with a higher stimulus intensity, pain and discomfort are increased, and its use is limited to appropriately assess muscle contractile properties. By accurately evaluating frequency properties in low‐intensity NMES, we can obtain force–frequency relationship data on people who have been considered contraindicated for stimulation at a high intensity (e.g., children, the advanced‐age elderly, and patients with disabilities). The aims of the present study were: (a) to examine the effect of the stimulus intensity on force–frequency and torque fluctuation–frequency relationships during NMES; and (b) to identify a novel parameter that can be used to evaluate muscle contractile properties.

We hypothesized that there is no significant difference between intensities regarding both force–frequency and torque fluctuation–frequency relationships, and that our novel index is more effective to investigate muscle contractile properties and suitable frequencies for each individual.

## 2 METHODS

### 2.1 Subjects

Nineteen healthy men (age: 28.6 ± 8.2 years, height: 171.5 ± 5.5 cm, weight: 65.1 ± 8.5 kg) volunteered for the study (35 legs). All subjects gave written informed consent for the study after receiving a detailed explanation of the purposes, potential benefits, and risks associated with participation. All subjects were healthy with no history of any musculoskeletal or neurological disorders. All study procedures were conducted in accordance with the Declaration of Helsinki and research code of ethics of Chukyo University, and were approved by the Committee for Human Experimentation of Chukyo University.

### 2.2 Experimental protocol

The subjects were comfortably seated in a custom‐made dynamometer (Takei Scientific Instruments Co., Ltd.) fixed to a force transducer (LU‐100KSE; Kyowa Electronic Instruments). The hip joint was flexed to 90° from an anatomical position, and testing was performed at a knee joint angle of 90°. Before NMES, subjects performed two MVCs at a knee joint angle of 90° with a ≥1‐min rest between trials. The MVC consisted of force rising (1–2 s), sustained (≥2 s), and relaxation (≤1 s) phases. To calculate the MVC torque during isometric contractions, the torque signal was sampled over 1 s during the sustained torque phase.

### 2.3 Neuromuscular electrical stimulation

**FIGURE 1:**
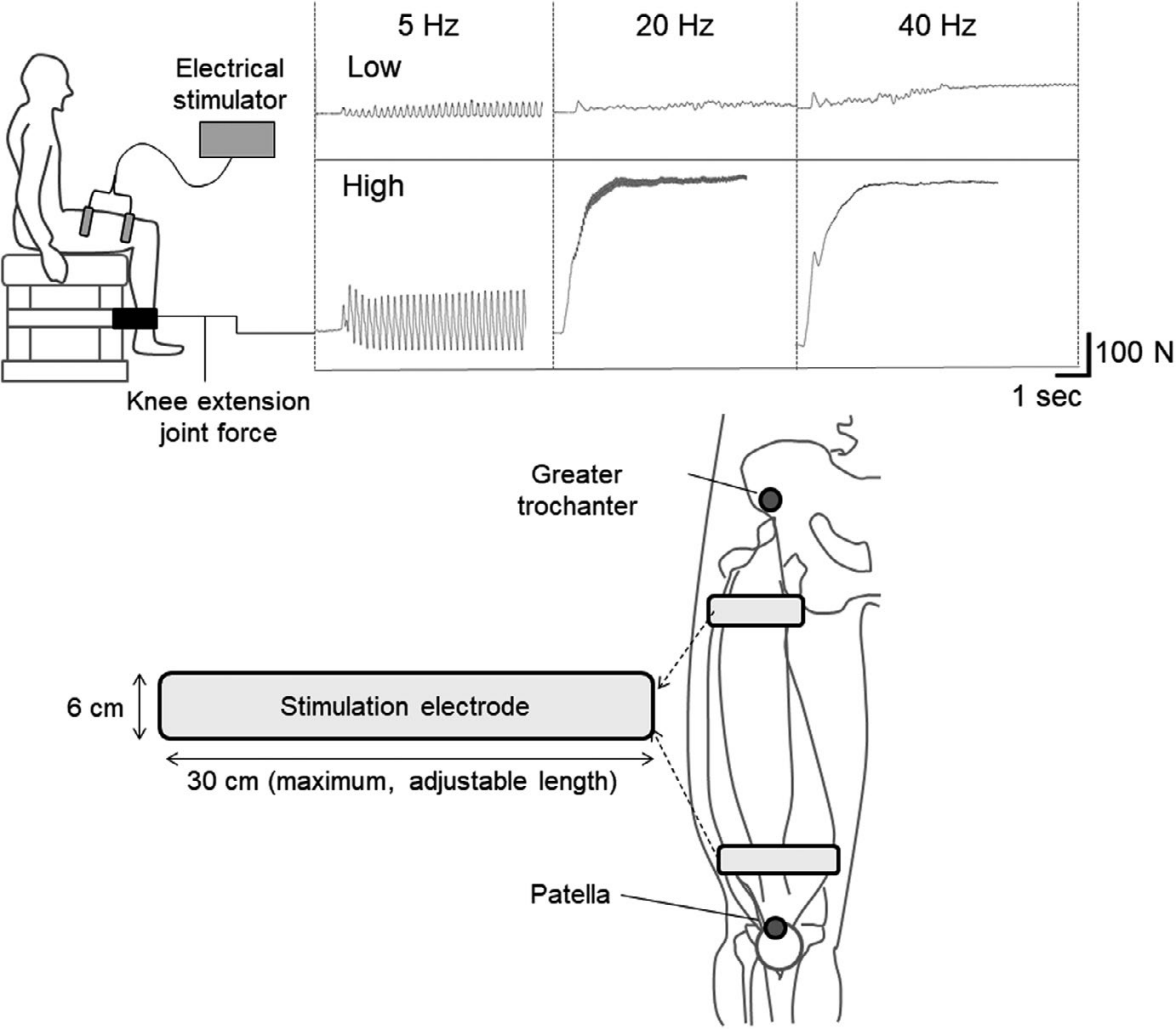
Experimental settings for neuromuscular electrical stimulation and examples of elicited force (upper), as well as electrode size and locations (lower)

The quadriceps femoris muscle was stimulated with two self‐adhesive electrodes (6 × 30 cm) located on proximal and distal parts of the anterior thigh using a custom‐made stimulator device (MTG Ltd.; Figure 1). One was attached on the distal part of the anterior thigh (proximal part of quadriceps tendon) and another one was attached on the proximal of the anterior thigh (near the greater trochanter). The electrode length could be adjusted depending on each subject's thigh so that it could appropriately cover the quadriceps femoris and be attached to the electric insulation sheet. The stimulation frequencies were set at 5, 10, 15, 20, 25, 30, and 40 Hz and the order was randomized. These stimulations were performed with ≥1‐min rest intervals between them. Biphasic square current pulses with a 100‐microsecond duration were applied. Electrical intensities of this device were 68 (low) and 113 (high) volts. The effective current at each frequency and intensity was as follows: low intensity: 2.20 mA at 5 Hz, 3.10 mA at 10 Hz, 3.88 mA at 15 Hz, 4.46 mA at 20 Hz, 4.98 mA at 25 Hz, 5.55 mA at 30 Hz, and 6.33 mA at 40 Hz; high intensity: 4.63 mA at 5 Hz, 6.10 mA at 10 Hz, 7.31 mA at 15 Hz, 8.11 mA at 20 Hz, 8.80 mA at 25 Hz, 9.39 mA at 30 Hz, and 10.04 mA at 40 Hz. Electrically elicited knee extension joint torque was calculated from the knee extension force measured by a dynamometer and each subject's leg length. Signals from the dynamometer were input into a computer running LabChart software at an analog‐digital conversion rate of 2,000 Hz (PowerLab; ADInstrument). The stimulation duration was <10 s and the last 3 s of the evoked torque were selected for each frequency and averaged for further analysis. We subsequently normalized the evoked torque at each frequency by the largest evoked torque (%peak torque). Furthermore, we extracted the frequency point of reaching tetanic contraction in two steps. First, this parameter was extracted from the force‐frequency curve. Evoked torque rose with increasing stimulus frequency, and it reached a plateau after tetanic contraction. Therefore, we extracted the frequency of the change in the inclination of the curve to minimum incline from ascent by the force‐frequency curve (FrT_TQ). Second, we differentiated evoked torque as torque fluctuation and normalized it at each frequency by the largest torque fluctuation. The torque fluctuation–frequency curve should decrease markedly and plateau with a change to tetanic contraction. Therefore, we extracted the frequency of the change in the inclination of the curve to minimum incline from descent by the torque fluctuation–frequency curve (FrT_FL).

### 2.4 Statistical analysis

The normality of all values was verified by the Shapiro–Wilk test. Evoked torque and fluctuation were compared between stimulus intensities (high and low) using two‐way ANOVA with repeated measures, respectively. A further post‐hoc test was used with a Bonferroni pairwise comparison to determine significant differences at each frequency. Similarly, the peak‐torque frequency and FrT at both intensities were also compared using two‐way ANOVA and the post hoc test with Bonferroni correction. The level of significance was set at *p* < .05 for all analyses. Statistical analyses were performed using SPSS statistics software (version 25.0J; IBM).

## 3 RESULTS

### 3.1 Frequency curve by NMES

**FIGURE 2:**
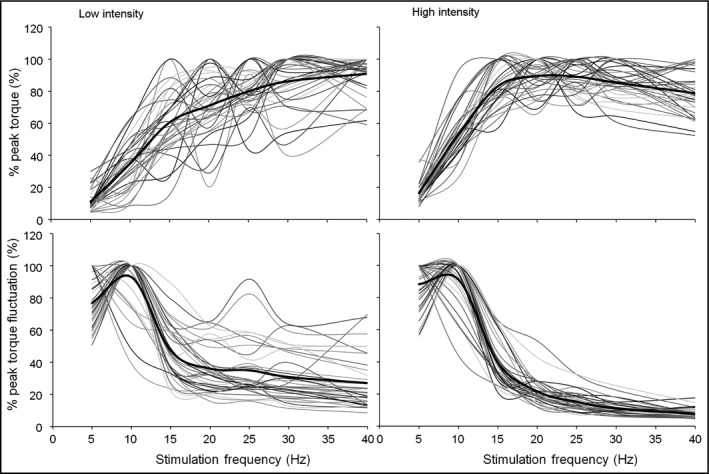
All individual data on the force–frequency (upper) and torque fluctuation–frequency (lower) relationships. Low intensity is on the left and high intensity is on the right. The bold black line shows the mean

Figure 2 shows force–frequency and torque fluctuation–frequency curves for all subjects at both intensities. The upper two graphs in Figure 2 show force‐frequency curves. At a low stimulus intensity, evoked torque increased progressively with an increasing stimulation frequency until 40 Hz. On the other hand, at a high stimulus intensity, evoked torque increased to around a 20–30‐Hz stimulation frequency; however, subsequently, it plateaued or decreased. Activation levels of maximal evoked torque were 18.0 ± 10.5% MVC with a low stimulus intensity and 42.0 ± 14.9% MVC with a high stimulus intensity. The lower two graphs in Figure 2 show torque fluctuation–frequency curves. At both stimulus intensities, torque fluctuation decreased markedly at around 20 Hz and decreased progressively or plateaued with an increasing stimulation frequency until 40 Hz.

### 3.2 Comparison by stimulus intensity

**FIGURE 3:**
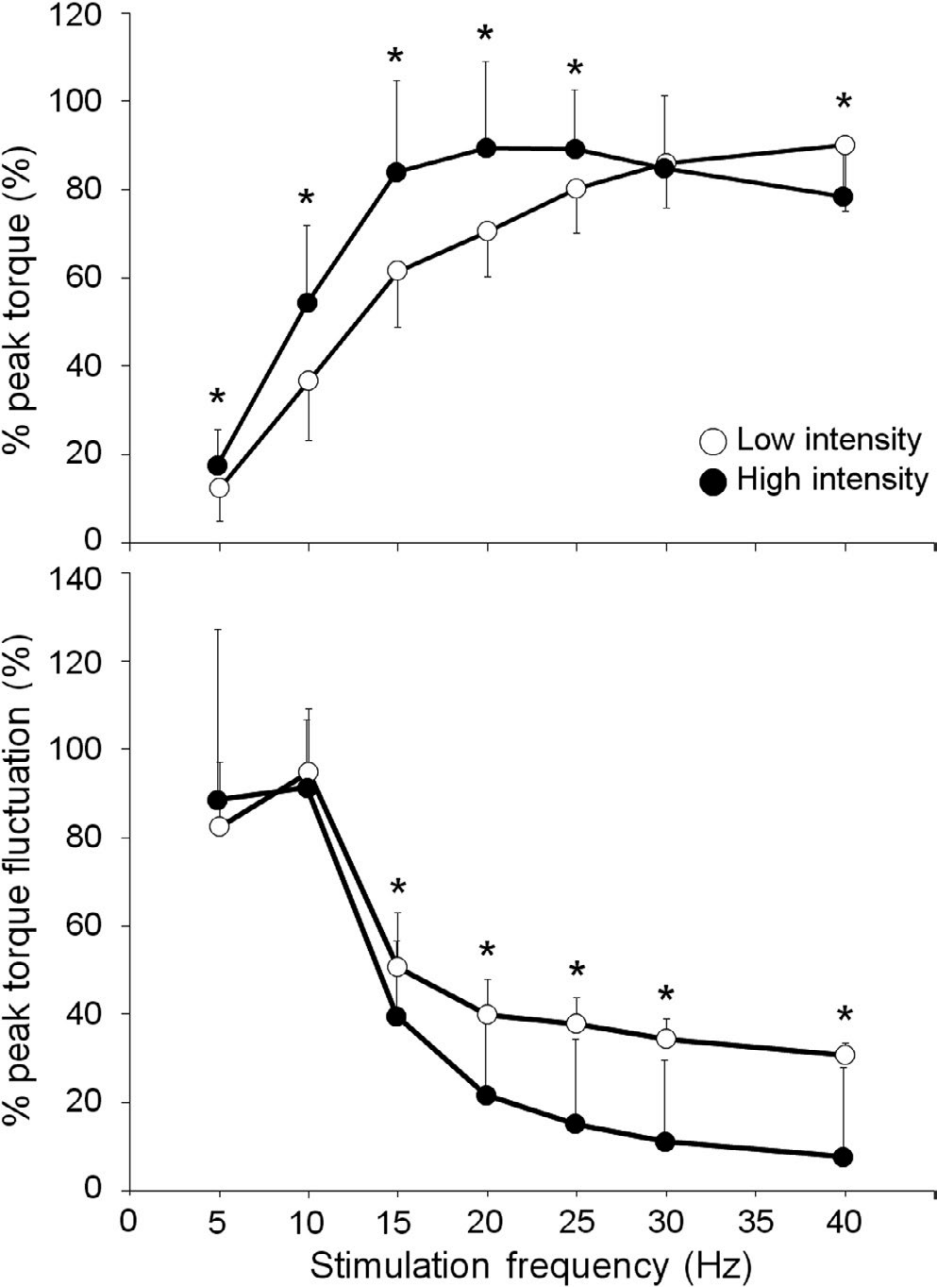
Comparison of force–frequency (upper) and torque fluctuation–frequency (lower) relationships between stimulus intensities. Values were normalized by the peak torque and peak torque fluctuation, respectively. The data are presented as the mean and standard deviation. *high versus low intensity, *p* < .05

There was a significant difference between stimulus intensities regarding the force–frequency relationship (*p* < .05; Figure 3). As the results of the post hoc test, %peak of the evoked torque with a high stimulus intensity was larger than that with a low stimulus intensity at 5, 10, 15, 20, 25, and 40 Hz. There was also a significant difference between stimulus intensities in the torque fluctuation–frequency relationship (*p* < .05; Figure 3). Furthermore, based on the post hoc test, %peak of the torque fluctuation at a high stimulus intensity was larger than that at a low stimulus intensity at 15, 20, 25, 30, and 40 Hz.

**FIGURE 4:**
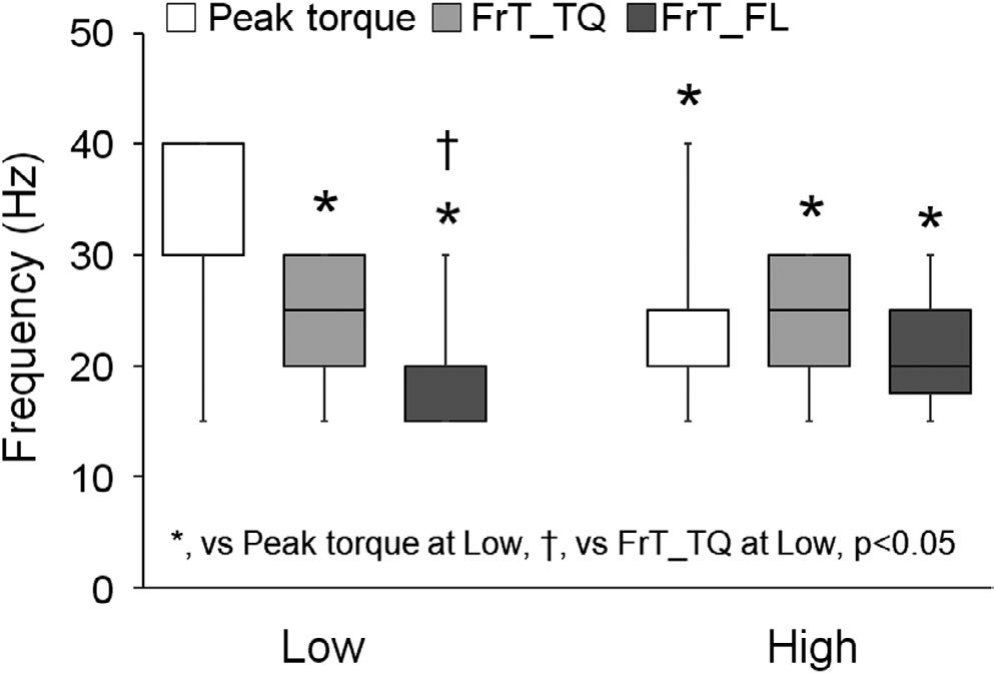
The peak‐torque frequency, extracted frequency point of reaching tetanic contraction from the force–frequency curve, and that from the peak torque fluctuation–frequency curve at both intensities. The data are presented as the median and percentile. FrT_FL, extracted frequency point of reaching tetanic contraction from torque fluctuation–frequency curve; FrT_TQ, extracted frequency point of reaching tetanic contraction from force–frequency curve. *versus peak‐torque frequency at low, ^†^versus extracted frequency point of reaching tetanic contraction from the force–frequency curve at a low intensity, *p* < .05

Figure 4 shows the peak‐torque frequency, FrT_TQ, and FrT_FL at both stimulus intensities. The peak‐torque frequency showed a significant difference between other parameters at both stimulus intensities (*p* < .05). For FrT_TQ and FrT_FL, there was no significant difference between stimulus intensities (*p* > .05). However, there was a significant difference between FrT_TQ and FrT_FL at a low stimulus intensity (*p* < .05).

## 4 DISCUSSION

The aims of the present study were: (a) to examine the effect of the stimulus intensity on force–frequency and torque fluctuation–frequency relationships during NMES; and (b) to identify a novel parameter to evaluate muscle contractile properties. The main findings were: (a) the stimulus intensity affected both the force–frequency and torque fluctuation–frequency relationships; (b) both relationships showed marked individual variation; (c) extracted FrT_TQ and FrT_FL showed no significant difference between stimulus intensities.

We demonstrated that the shapes of force–frequency curves at low and high stimulus intensities varied. During NMES, the recruitment pattern does not follow Henneman's size principle, whereby voluntary motor units are recruited progressively from smallest to largest (Henneman et al., 1965). The motor unit activation order during NMES is nonselective, and recruited motor units are not related to fiber types (Binder‐Macleod et al., 1995; Gregory & Bickel, 2005; Jubeau et al., 2007). Gregory and Bickel (2005) reviewed the recruitment pattern during NMES, and reported that motor units are activated without clear arrangement. Binder‐Macleod et al. (1995) determined the twitch and force–frequency relationships at different force levels during transcutaneous electrical stimulation. As a result, twitch contractile speeds did not differ between evoked intensities equal to 20% MVC, 50% MVC, and 80% MVC. They suggested that the relative proportions of fast and slow motor units at different stimulus intensities would be constant. The activation pattern of motor units would not have changed with the stimulus intensity because the recruitment order of motor units was randomized. However, in contrast to this theory, Sinacore et al. (1990) suggested that NMES selectively activated type II muscle fibers. They showed that glycogen depletion in skeletal muscle was markedly greater in type II muscle fibers after NMES in the quadriceps femoris muscle. Furthermore, Greenhaff et al. (1993) reported that energy levels made available by the glycolysis system were two‐times higher than in slow muscle fibers. The energy metabolism properties, unlike voluntary contraction by the selective recruitment of fast muscle fibers (type II), were suggested by electrical stimulation. We noted a difference cause by the stimulus intensity in the force‐frequency relationship. Although the main theory regarding the recruitment pattern by NMES states that it is nonselective and random (Maffiuletti, 2010), the present results support Greenhaff et al. (1993) and Sinacore et al. (1990). In other words, the present study followed the results of Binder‐Macleod et al. (1995) and supported their suggestion regarding our novel parameter.

Natsume et al. (2018) conducted a study on the effect of 8‐week NMES training with low and high intensities (both intensities were below 60% MVC). They showed the hypertrophy of both muscle fiber types (types I and II) at both intensities, although a high intensity led to a higher rate of hypertrophy than a low intensity. Their study indicated that both slow and fast muscle fibers could be recruited even at a low intensity during NMES. It is considered that motor unit recruitment is not altered depending on the evoked force level. Their set‐up intensities throughout the entire training period at high and low intensities were approximately 63% MVC and 33% MVC, respectively. In contrast, average contraction levels at high and low intensities were 42% MVC and 18% MVC, respectively, even at the frequency evoking peak torque in the present study. As they also mentioned in terms of the recruitment motor unit type, whether a training intensity less than 30% MVC can promote muscle hypertrophy remains unknown. In another study, Conwit et al. (1999) reported that the motor unit firing rate was increased appreciably at an intensity above 30% MVC. Thus, there is a possibility that the relationship between the evoked force level and motor unit recruitment threshold is an important factor, and the cut‐off intensity might be 20%–30% MVC.

Muscle contractions induce Ia, Ib, and other afferent inputs to motor neurons due to alterations in lengths of muscle fibers, muscle spindles, ligaments, and other tissues (Dyhre‐Poulsen & Krogsgaard, 2000; Hultborn et al., 1987; Babault et al., 2003). These neural reflex circuits would be active even during electrically elicited isometric contractions that we employed in this study. Also, ascending afferent inputs to sensorimotor cortical areas by NMES have been reported, and this is supported by neural adaptations following chronic NMES (Collins et al., 2002; Dean et al., 2008; Hortobagyi & Maffiuletti, 2011). Moreover these effects would depend on parameters of NMES such as the pulse width or frequency (Collins, 2007; Martin et al., 2016). We therefore considered that differences in force‐frequency and torque fluctuation‐frequency relationships and their different responses between low and high stimulation intensities could be partly caused by these neural factors, but it would be difficult to quantify their effects on our results.

Based on the present results, there is a possibility that the stimulatable regions of the quadriceps femoris muscle were dependent on the stimulus intensity. Binder‐Macleod et al. (1995) did not identify differences in the force‐frequency relationship between conditions equal to 20% MVC and 50% MVC, while only a slight shift to the left was observed at 80% MVC and a 20–70‐Hz stimulus intensity. They reported that the stimulated field was made up of superficial muscle (e.g., rectus femoris) with low intensities and deep muscle (e.g., vastus intermedius) with high intensities. Adams et al. (1993) mapped the activation pattern after evoked isometric contractions of the quadriceps femoris muscle during NMES using magnetic resonance images. They suggested that even at low force levels, deep muscle fibers could be recruited, even those next to the femur, such as the vastus intermedius. This finding provides evidence that the recruitment pattern during NMES is nonselective and random. They also stated that the reversal of the motor unit recruitment pattern can be explained by the linear relationship between the evoked torque and cross‐sectional area of the quadriceps femoris muscle showing increased contractile activity after stimulation. Therefore, the present results support Binder‐Macleod's suggestion, whereby deeper muscle was stimulated at a high intensity and superficial muscle was stimulated at a low intensity, and can be explained by Adam's results regarding the linear relationship.

Evoked torque is an important factor because the frequency is directly correlated with torque production (Glaviano & Saliba, 2016). The peak‐torque frequency simply showed this change in torque production by increasing the stimulus frequency. As a result, the peak‐torque frequency showed a significant difference between stimulus intensities. It was considered that this is caused by incomplete tetanic contraction with a low stimulus intensity. This was also shown by the results of the torque fluctuation‐frequency relationship. The torque fluctuation at each frequency normalized by the largest torque fluctuation at a low stimulus intensity was significantly larger than that at a high stimulus intensity. This meant that the torque fluctuation did not decline completely, and suggested that the contraction pattern did not reach the tetanic contraction phase with a low stimulus intensity. It was suggested that the non‐tetanic contraction pattern was associated with the result that evoked torque at 40 Hz was the highest, continuing to increase with a rise in the stimulation frequency, and variation of the peak‐torque frequency was large at a low stimulus intensity.

The force‐frequency curve was extracted using the change of evoked torque with an increasing stimulus frequency. Moreover we considered that the change in torque fluctuation is also important as a factor showing the change in the contraction pattern. In other words, it was considered that the torque fluctuation continues to decrease until the contraction pattern shows complete tetanic contraction, and it plateaus after this point. Therefore, we calculated FrT_FL. Neither FrT_TQ nor FrT_FL showed a significant difference between stimulus intensities. The peak‐torque frequency, which was influenced by the stimulus intensity, showed a significant difference from both FrT_TQ and FrT_FL at both low and high stimulus intensities. These results may be considered effective parameters at a glance regardless of the stimulus intensity. However, there was a significant difference between FrT_TQ and FrT_FL at a low stimulus intensity. From this result, there is a possibility that FrT_FL is influenced by the stimulus intensity. As shown in Figures 2 and 4, there were individual variations in the peak‐torque frequency for FrT_TQ and FrT_FL. The force‐frequency curve exhibits changeable behavior (Binder‐Macleod et al., 1998), the optimal frequency for training or rehabilitation is also changeable, and suitable frequencies for individuals are considered to be different. Glaviano and Saliba (2016) reported that the optimal frequency for training is 30–50 Hz. They mentioned that this range of stimulus frequency can improve torque production, minimize fatigue, and improve patient comfort levels. In this study, FrT_TQ showed similar results. Moreover FrT_FL was also applicable at a high intensity (42% MVC of maximum). We suggest that these parameters could be applied to appropriately extract the “optimal” frequency for individuals.

In conclusion, the present study provides a novel perspective to evaluate contractile properties based on NMES and demonstrated a difference in those parameters between high and low stimulus intensities. Our findings support the theory that NMES selectively activates Type II fibers, as mentioned by Binder‐Macleod et al. (1995), in contrast to the theory that NMES induces nonselective motor unit recruitment without a sequential order (Gregory & Bickel, 2005). We suggest that the stimulus intensity should be considered on investigating the force–frequency relationship during NMES in the quadriceps femoris muscle. However, we also suggest that we can simultaneously evaluate contractile properties using a novel parameter, FrT_TQ or FrT_FL. Moreover the torque fluctuation‐frequency relationship was indicated as an original parameter to discuss contractile properties. This novel parameter may lead to a new method to assess the torque fluctuation‐frequency relationship at the point of tetanic contraction and become an effective index to investigate the optimal stimulation frequency.

## CONFLICT OF INTEREST

Author Shuhei Kawade was employed by the company MTG CO., Ltd. The remaining authors declare that the research was conducted in the absence of any commercial or financial relationships that could be construed as a potential conflict of interest. The authors declare that this study received funding from MTG Co., Ltd. The funder had the following involvement with the study: They provided equipment used in this study but were not involved in data collection or analysis.

## AUTHOR CONTRIBUTION

A.T. contributed to planning research, conducting experiment, analyzing data, writing the manuscript. S.K. contributed to designing and helping experiment, discussing results, and editing and reviewing the manuscript. T.M. contributed to planning research, discussing results, and editing and reviewing the manuscript. K.W. contributed to planning research, designing and helping experiment, discussing results, and writing and editing the manuscript.

## ETHICAL APPROVAL

All procedures performed in studies involving human participants were conducted in accordance with the ethical guidelines of Chukyo University (Committee for Human Experimentation of Chukyo University, 2017‐002) and the 1964 Helsinki declaration and its later amendments or comparable ethical standards.

## Data Availability

The data that support these findings are available upon reasonable request from the corresponding author.
